# Clinical analysis of generalized pustular psoriasis in Chinese patients: A retrospective study of 110 patients

**DOI:** 10.1111/1346-8138.15958

**Published:** 2021-05-21

**Authors:** Jianfeng Zheng, Wenjuan Chen, Yunlu Gao, Fujuan Chen, Ning Yu, Yangfeng Ding, Na Liu

**Affiliations:** ^1^ Department of Dermatology Shanghai Skin Disease Hospital Institute of Psoriasis Tongji University School of Medicine Shanghai China

**Keywords:** acitretin, generalized pustular psoriasis, glucocorticoids, methotrexate, tumor necrosis factor inhibitor

## Abstract

Generalized pustular psoriasis is an immune‐mediated dermatologic condition characterized by widespread, sterile, subcorneal pustules. However, limited information exists regarding the clinical course of generalized pustular psoriasis. This study aimed to examine the precipitating factors, clinical manifestations, laboratory data, relapse patterns, and prognosis of generalized pustular psoriasis at our hospital and to improve the diagnosis and treatment. A retrospective analysis was conducted for generalized pustular psoriasis in our department from 2014 to 2019. In total, 110 patients were included in our study (mean age 46.5 years). The female:male ratio was 1:2.7. Ninety‐four (85.5%) had a psoriasis vulgaris history, 12 (10.9%) had a psoriatic arthritis history, five (4.5%) had an erythrodermic psoriasis history, and 16 (14.5%) had a family history of psoriasis. Eleven (10.0%) cases were triggered by infections and 17 (15.5%) were caused by the sudden discontinuation of systemic drugs. During hospitalization, the proportion of patients with hyperlipidemia was higher after acitretin treatment than before acitretin treatment (*P *< 0.05). The proportion of patients with abnormal liver function was higher after methotrexate treatment than before methotrexate treatment (*P *> 0.05). The onset age of generalized pustular psoriasis was younger in patients without prior psoriasis (*P *< 0.05). The mean time to pustular clearance was shorter in patients with prior psoriasis than in those without prior psoriasis (*P *> 0.05). Moreover, among patients with fever, skin lesion clearance rates were highest in the biological agent group (81.8%). However, among patients without fever, skin lesion clearance rates were highest in the acitretin group (86.7%). No patients presented serious complications or died. Our study presents the detailed clinical course of generalized pustular psoriasis in Chinese patients. These results will help to better understand and treat generalized pustular psoriasis.

**Abbreviations**
CRP: C‐reactive protein
EP: erythrodermic psoriasis
ESR: elevated erythrocyte sedimentation rate
GPP: generalized pustular psoriasis
MTX: methotrexate
PsA: psoriatic arthritis
PsV: psoriasis vulgaris
SD: standard deviation
TNF: tumor necrosis factor

## 1 INTRODUCTION

Psoriasis is a chronic, immune‐mediated, inflammatory skin disease that is not uncommon in adults and affects 1%–3% of the global population.^1^ In particular, generalized pustular psoriasis (GPP) is a rare variant of psoriasis that is characterized by the widespread eruption of sterile, subcorneal pustules and epidermal scaling accompanied by signs of toxicity, fever, and leucocytosis. GPP has a significant negative impact on the quality of life and is even potentially life‐threatening in some severe cases. Few publications have described the clinical course of GPP in Chinese patients; previous publications include a report in which Wang et al. studied the clinical features of GPP in 26 pediatric patients.^2^ Despite its heterogeneity, it is important to recognize the overall clinical course of GPP for an accurate prognosis of the disease. In this study, we retrospectively investigated the clinical characteristics, laboratory examinations, and therapeutic and systemic effects of 110 GPP patients treated at the dermatology unit of our hospital over the past 6 years.

## 2 METHODS

A retrospective study was conducted in patients with GPP admitted to Shanghai Skin Disease Hospital in China from January 2014 to December 2019. All data used were from inpatient medical records. GPP was diagnosed based on history, characteristic skin lesions, and related laboratory examinations that met the international criteria^3^ and was confirmed by at least two dermatologists. The clinical features and course were analyzed according to the presence of previous plaque psoriasis and body temperature. Characteristic information including age, sex, the age of onset, onset season, family history of psoriasis, previous history of psoriasis vulgaris (PsV)/psoriatic arthritis (PsA)/erythrodermic psoriasis (EP), inducing factor, laboratory data, treatments, and hospitalization days was obtained from each patient. This study was approved by the research ethics committee of Shanghai Skin Disease Hospital.

## 3 RESULTS

A total of 110 patients, which included 80 men and 30 women, were included in the study. The clinical features of the 110 patients are detailed in Tables 1 and 2. The age of pustule onset ranged from birth to 79 years old, with a mean age of 43.4 years. Among them, 13 (11.8%) patients had the first episode of von Zumbusch type GPP by 18 years of age, 40 (36.4%) patients by 18–45 years old, and 57 (51.8%) at over 45 years old. Disease episodes occurred more frequently in autumn (32.7% of patients). Sixteen patients had a family history of psoriasis and 94 had a previous history of PsV. A history of PsA (12 patients) and EP (five patients) was only observed in patients with a previous history of PsV. Forty‐six patients had nail damage, including thickened nails and thimble nails. Scalp involvement was observed in 59 patients, with three cases accompanied by pustules. Details of the aggravating factors in 39 patients are follows: infection in 11, oral Chinese medication in three and drug withdrawal in 17, along with other causes. Infection included nine cases of upper respiratory infection, one case of tonsillitis, and one case of erysipelas. However, no patients without a previous history of PsV experienced infections. Medication withdrawal included four cases of acitretin, four cases of methotrexate (MTX), two cases of biological agents, four cases of corticosteroids, and three cases of Chinese medication. The skin lesion started on the trunk in nine patients, on the face and neck in one patient, and on the limbs in 27 patients. Seventy‐four patients presented generalized lesions from the beginning. The mean hospitalization time of all patients was 16.2 ± 6.5 days. The pustules completely resolved in 94 patients and the mean time was 9.3 ± 6.6 days. The mean time for pustular clearance in patients with a previous history of PsV was shorter than that in patients without a previous history of PsV (8.9 ± 5.5 vs 11.9 ± 10.8 days, *P *> 0.05). During hospitalization, abnormal laboratory values were typical, such as elevated erythrocyte sedimentation rate (ESR) (50%), hypoalbuminemia (63.6%), hypocalcemia (40.2%), leucocytosis (51.4%), abnormal liver function (16.4%), hyperlipidemia (19.1%), and elevated hypersensitive CRP (90%). The rate for achieving skin lesion clearance was highest in patients treated with biological agents (80%) and second highest in patients treated with corticosteroids (75%). However, there was no significant difference between the two groups concerning the previous history of PsV (*P *> 0.05).

**TABLE 1:** Clinical features of patients with GPP with or without previous PsV

	Total	History of PsV		*P* *
		With PsV	Without PsV	
Total number of patients (F/M)	110 (30/80)	94 (22/72)	16 (8/8)	–
Age (mean SD, years)	46.0 ± 18.4	47.0 ± 18.2	40.3 ± 18.8	0.202
Age of onset (mean SD, years)	34.1 ± 18.1	34.7 ± 18.0	30.6 ± 18.5	0.419
Age of pustule onset (mean SD, years)	43.4 ± 19.2	45.5 ± 18.7	31.3 ± 18.1	0.009
Age, n (%)				
<18 years old	13 (11.8%)	9 (9.57%)	4 (25%)	–
18‐45 years old	40 (36.4%)	34 (36.17%)	8 (50%)	–
>45 years old	57 (51.8%)	51 (54.26%)	4 (25%)	–
Seasons, n (%)				
Spring	27 (24.5%)	22 (23.4%)	5 (31.25%)	–
Summer	30 (27.3%)	26 (27.7%)	6 (37.5%)	–
Autumn	36 (32.7%)	30 (31.9%)	4 (25%)	–
Winter	17 (15.5%)	16 (17.0%)	1 (6.25%)	–
Involvement of scalp, n (%)	59 (53.6%)	54 (57.4%)	5 (31.3%)	0.052
Involvement of nails, n (%)	46 (41.8%)	43 (45.7%)	3 (18.8%)	0.043
History of EP, *n* (%)	5 (4.5%)	5 (5.3%)	0	–
History of PsA, n (%)	12 (10.9%)	12 (12.8%)	0	–
Family history of psoriasis, n (%)	16 (14.5%)	13 (13.8%)	3 (18.8%)	0.606
Fever, n (%)	58 (52.7%)	49 (52.1%)	9 (56.3%)	0.76
Aggravating factors, n				
Infection	11	11	0	–
Medications	3	2	1	–
Overwork	8	8	0	–
Drug withdrawal				
(Acitretin/MTX/biological agents/steroids/traditional Chinese medication)	4/4/2/4/3	3/4/1/3/1	1/0/1/1/2	–
No apparent triggers	57	44	13	–

EP, erythrodermic psoriasis; F, female; GPP, generalized pustular psoriasis; M, male; MTX, methotrexate; PsA, psoriatic arthritis; PsV, psoriasis vulgaris; SD, standard deviation.

* *P* values represent a comparison between GPP patients with a history of plaque psoriasis vs GPP patients without a history of plaque psoriasis. *P *< 0.05 is considered statistically significant.

**TABLE 2:** Clinical features of patients with GPP with or without previous PsV

	Total	History of PsV		*P* *
		With PsV	Without PsV	
Total number of patients (F/M)	110 (30/80)	94 (22/72)	16 (8/8)	–
Mean hospitalization time (mean SD, days)	16.2 ± 6.5	16.2 ± 5.7	16.1 ± 10.2	0.971
Mean time for body temperature returned to normal (mean SD, days)/Total number of patients (n)	5.8 ± 5.7 (53)	5.7 ± 5.3 (44)	6.6 ± 7.7 (9)	0.758
Mean time for pustule clearance (mean SD, days)/Total number of patients (n)	9.3 ± 6.6 (94)	8.9 ± 5.5 (80)	11.9 ± 10.8 (14)	0.328
Results, n (%)				
Pustule clearance	94	80	14	–
Partial pustule clearance	15	14	1	–
Temporary discharge	1	0	1	–
Laboratory findings, n/n tested (%)				
Elevated ESR	46/92 (50%)	40/79 (50.6%)	6/13 (46.2%)	0.765
Hypoalbuminemia	70/110 (63.6%)	62/94 (66.0%)	8/16 (50.0%)	0.22
Hypocalcemia	41/102 (40.2%)	36/87 (41.4%)	5/15 (33.3%)	0.557
Leucocytosis	56/109 (51.4%)	46/93 (49.5%)	10/16 (62.5%)	0.335
Abnormal liver function	18/110 (16.4%)	16/94 (17.0%)	2/16 (12.5%)	0.651
Hyperlipidemia	21/110 (19.1%)	16/94 (17.0%)	5/16 (31.3%)	0.181
Elevated hypersensitive CRP	45/50 (90%)	36/40 (90%)	9/10 (90%)	1
Treatment, effective rate (%)				
Acitretin	70/98 (71.4%)	61/84 (72.6%)	9/14 (64.3%)	0.523
MTX	5/8 (62.5%)	5/7 (71.4%)	0/1 (0%)	0.168
Biological agents	12/15 (80.0%)	11/14 (78.6%)	1/1 (100%)	0.605
Steroids	6/8 (75.0%)	2/3 (66.7%)	4/5 (80.0%)	0.673

CRP, C‐reactive protein; ESR, erythrocyte sedimentation rate; F, female; GPP, generalized pustular psoriasis; M, male; MTX, methotrexate; PsV, psoriasis vulgaris; SD, standard deviation.

* *P* values represent a comparison between GPP patients with a history of plaque psoriasis vs GPP patients without a history of plaque psoriasis. *P* < 0.05 is considered statistically significant.

In our study, 58 patients suffered from fever and their body temperature was 38‐41ºC. Forty‐three patients underwent blood culture tests, which were all negative. In 53 patients, the mean time for body temperature to return to normal was 8.71 ± 4.58 days. The body temperature of the remaining five patients did not return to normal before discharge. Compared with patients with fever, the mean time for pustular clearance was shorter in patients without fever (11.1 ± 7.4 vs 7.4 ± 5.0, *P *< 0.05). The proportion of patients with hypoalbuminemia, hypocalcemia, leucocytosis, and abnormal liver function was higher in patients with fever than in patients without fever (77.6% vs 48.1%, *P *< 0.05; 52.7% vs 25.5%, *P *< 0.05; 69.0% vs 31.4%, *P *< 0.05; 22.4% vs 9.6%, *P *> 0.05). Moreover, in patients with fever, the rate of achieving skin lesion clearance was the highest in the group treated with biological agents (81.8%). However, in patients without fever, the rate of achieving skin lesion clearance was the highest in the acitretin group (86.7%). These data are shown in Table 3.

**TABLE 3:** Clinical features of GPP patients with or without fever

	Total	Body temperature		*P* *
		With fever	Without fever	
Total number of patients (F/M)	110 (30/80)	58 (12/46)	52 (18/34)	–
Age (mean SD, years)	46.0 ± 18.4	47.6 ± 18.1	44.3 ± 18.7	0.351
Age of pustule onset (mean SD, years)	43.4 ± 19.2	44.3 ± 19.4	42.5 ± 19.2	0.627
Mean hospitalization time (mean SD, days)	16.2 ± 6.5	17.2 ± 7.4	15.1 ± 5.0	0.071
Mean time for pustule clearance (mean SD, days)/Total number of patients (n)	9.3 ± 6.6 (94)	11.1 ± 7.4 (48)	7.4 ± 5.0 (46)	0.005
Results, n (%)				
Pustule clearance	94	48	46	–
Partial pustule clearance	15	9	6	–
Temporary discharge	1	1	0	–
Laboratory findings, n/n tested (%)				
Elevated ESR	46/92 (50%)	19/46 (41.3%)	27/46 (58.7%)	0.095
Hypoalbuminemia	70/110 (63.6%)	45/58 (77.6%)	25/52 (48.1%)	0.001
Hypocalcemia	41/102 (40.2%)	29/55 (52.7%)	12/47 (25.5%)	0.005
Leucocytosis	56/109 (51.4%)	40/58 (69.0%)	16/51 (31.4%)	0.000
Abnormal liver function	18/110 (16.4%)	13/58 (22.4%)	5/52 (9.6%)	0.07
Hyperlipidemia	21/110 (19.1%)	16/94 (17.0%)	5/16 (31.3%)	0.181
Elevated hypersensitive CRP	45/50 (90.0%)	27/28 (96.4%)	18/22 (81.8%)	0.087
Treatment, effective rate (%)				
Acitretin	70/98 (71.4%)	31/53 (58.5%)	39/45 (86.7%)	0.002
MTX	5/8 (62.5%)	2/4 (50%)	¾ (75%)	0.465
Biological agents	12/15 (80.0%)	9/11 (81.8%)	3/4 (75%)	0.77
Steroids	6/8 (75.0%)	6/8 (75%)	0/0 (0%)	–

CRP, C‐reactive protein; ESR, erythrocyte sedimentation rate; F, female; GPP, generalized pustular psoriasis; M, male; MTX, methotrexate; SD, standard deviation.

* *P* values represent a comparison between GPP patients with fever vs GPP patients without fever. *P *< 0.05 is considered statistically significant.

As shown in Table 4, 98 patients were treated with systemic acitretin and the dose ranged from 0.5 to 1.0 mg/kg/day (the maximum dose did not exceed 40 mg/day). However, 19 patients were switched to other treatments because of treatment failure, one patient was discharged temporarily due to stomach discomfort, and eight patients still had a few pustules or scabs on the day of discharge. The mean time for body temperature to return to normal was 5.7 ± 6.0 days, the mean hospitalization time was 15.4 ± 5.6 days, and the mean time for pustule clearance was 8.5 ± 5.2 days. During hospitalization, the proportion of patients with hyperlipidemia was higher after treatment than before treatment (47.3% vs 22.8%, *P *<0.05). Seven patients were treated with MTX, and the dose ranged from 7.5 to 15 mg/week. With the exception of two patients who still had a few pustules on the day of discharge, five patients achieved a satisfactory clinical response. The mean time for pustule clearance was 5 ± 4.4 days, the mean time for body temperature to return to normal was 2.4 ± 4.34 days, and the mean hospitalization time was 16.4 ± 7.7 days. The immediate efficacy of tumor necrosis factor inhibitors in controlling acute GPP attacks was evaluated in 15 patients. Three patients had a few pustules on the day of discharge and 12 patients achieved a satisfactory clinical response with a mean time for pustular clearance of 11.4 ± 6.2 days. In the patient who received infliximab treatment, the time to pustular remission was 2 weeks. In the patient who received adalimumab with acitretin, pustular remission was achieved in 11 days. One patient was treated with etanercept, and the time to pustular remission was 7 days. Nine patients received etanercept combined with acitretin. One patient was discharged early because of financial problems, and eight patients achieved a satisfactory clinical response, with a mean time to pustular clearance of 12.6 ± 7.1 days. Three patients were treated with etanercept with MTX. One patient left the hospital without pustular remission due to poor efficacy, and two patients achieved a satisfactory clinical response, with a median time to pustule clearance of 9 days (range 5–13 days). No patients presented serious complications or died.

**TABLE 4:** Clinical features of GPP patients treated with different medications

	Treatment			
	Acitretin	MTX	Biological agents	Steroids
Mean hospitalization time (mean SD, days)	15.4 ± 5.6	18.1 ± 7.2	15.1 ± 5.0	20.5 ± 12.4
Mean time for body temperature returned to normal (mean SD, days)/Total number of patients (n)	5.7 ± 6.0 (32)	5 ± 4.4 (3)	4.5 ± 2.5 (11)	8.9 ± 7.9 (7)
Mean time for pustule clearance (mean SD, days)/Total number of patients (n)	8.5 ± 5.2 (70)	5 ± 4.4 (5)	11.4 ± 6.2 (12)	12.9 (6)
Results, n				
Pustule clearance	70	5	12	6
Partial pustule clearance	9	2	3	2
Treatment failure	19	1	0	0
Laboratory findings before treatment, n/n tested (%)				
Leucocytosis	34/78 (43.6%)	3/7 (42.9%)	12/15 (80.0%)	7/8 (87.5%)
Hypoalbuminemia	46/79 (58.2%)	3/7 (42.9%)	15/15 (100%)	5/8 (62.5%)
Abnormal liver function	14/79 (17.7%)	2/7 (28.6%)	1/15 (6.7%)	1/8 (12.5%)
Hyperuricemia	18/79 (22.8%)	4/7 (57.1%)	4/15 (26.7%)	0/8 (0%)
Hyperlipidemia	18/79 (22.8%)	1/7 (14.3%)	1/15 (6.7%)	1/8 (12.5%)
Elevated hypersensitive CRP	31/33 (93.9%)	5/6 (83.3%)	2/2 (100%)	6/6 (100%)
Hypocalcemia	27/72 (37.5%)	0/7 (0%)	9/14 (64.3%)	4/8 (50.0%)
Elevated ESR	17/66 (25.8%)	1/5 (20.0%)	7/13 (53.8%)	3/7 (42.9%)
Laboratory findings after treatment, n/n tested (%)				
Leucocytosis	13/66 (19.7%)	1/7 (14.3%)	3/12 (25.0%)	4/5 (80.0%)
Hypoalbuminemia	35/66 (53.0%)	1/7 (14.3%)	10/12 (83.3%)	2/5 (40.0%)
Abnormal liver function	11/66 (16.7%)	4/7 (57.1%)	0/12 (0%)	2/5 (40.0%)
Hyperuricemia	15/66 (22.7%)	3/7 (42.9%)	3/12 (25.0%)	2/5 (40.0%)
Hyperlipidemia	26/55 (47.3%)	0/3 (0%)	1/6 (16.7%)	2/4 (50.0%)
Elevated hypersensitive CRP	9/1 9(47.4%)	3/5 (60.0%)	2/3 (66.7%)	1/3 (33.3%)
Hypocalcemia	1/7 (14.3%)	0/0 (0%)	0/1 (0%)	0/1 (0%)

CRP, C‐reactive protein; GPP, generalized pustular psoriasis; MTX, methotrexate; SD, standard deviation.

## 4 DISCUSSION

In our study, the disease started at a mean age of 43.4 years, which is similar to the 40–60 year age range reported by Baker and Jin.^4^, ^5^ The mean onset age of pediatric patients was 10.2 years, which is slightly higher than the age of 6.9 years that Wei Liu observed in his report.^2^ In previous studies, GPP more frequently occurred in women, and Youn et al.^6^ reported a male:female ratio of 1:2. In this study, the male:female ratio was 2.7:1, which revealed that the proportion of men in our study was considerably higher than that in previous studies. However, our study showed that the incidence in male children was slightly higher than that in female children, which was in accordance with other published reports.^7^ Our study showed that GPP occurred more frequently in summer and autumn, which was different from PsV. A previous study reported that 10.7% of GPP patients had a history of plaque psoriasis.^3^ However, the percentage of patients with a history of plaque psoriasis was 85.5% in this study population. In cases of acute GPP, Youn et al.^6^ reported that fever was associated in 62.5% of patients. In our study, the rate of fever in patients (52.7%) was similar. There were numerous triggering factors, including infections, the untimely withdrawal or tapering of oral medications, operations, pregnancy, medications, mental stress, and climate, but some of these factors were not obvious. In our patients, attacks were mostly induced by untimely withdrawal or decrease of oral medications and infections. However, more than 50% of patients had no apparent triggers, especially in patients without a previous history of PsV (81.2%).

There is a variety of drugs and regimens for the treatment of GPP, including retinoids, glucocorticoids, immunosuppressive drugs, and photochemical therapy. Tretinoin has been used as a first‐line drug for the treatment of GPP according to the guidelines. Retinoids (especially acitretin) can regulate epithelial cell proliferation and differentiation, and affect immune system function and inflammatory processes. In this dataset, 97 patients (88.2%) were treated with acitretin. Among them, treatment was effective in 70 patients, and the total efficacy rate was 71.4%, which was slightly lower than the 83.7% efficacy rate reported by Teng Jiayong.^8^ The proportion of patients who showed pustular clearance was higher in patients without fever than in patients with fever, which proved that body temperature could affect the efficacy of acitretin. At present, the use of biological agents is becoming a new option for GPP. In 2018, Morita et al. found that biological agents were very effective and well tolerated in the treatment of Japanese patients with GPP.^9^ In this study, 15 patients were treated with a tumor necrosis factor inhibitor, the total efficacy rate was 80%, the mean time to pustular clearance was 11.4 ± 6.2 days, and the mean hospitalization time was 15.1 ± 5.0 days. However, compared with the acitretin group, there was no significant difference in the biological agent group (*P *> 0.05), therefore more data are needed to confirm the advantages of biological agents.

The specific pathogenesis of GPP is still unclear, and it has been considered a subtype of psoriasis for a long time. GPP can occur in people with or without a history of PsV, so it has been considered to have a certain association with PsV. As we know, Th17 cells have been described as a central mediator of the immune pathogenesis of psoriasis.^10^ However, Johnston et al. demonstrated a significant upregulation of IL‐17A in GPP. Furthermore, IL‐23, the maintenance factor of IL‐17 production, which is highly expressed in psoriasis,^11^ is also expressed in GPP.^12^ Thus, the IL‐23/Th17 axis also seems to play an important role in GPP, which may be the reason why there is no statistical significance of the mean time for pustular clearance between GPP with PsV and GPP without PsV. From a genetic point of view, some cases had a family history, whether in PsV or pustular psoriasis, so genetics may play a role in the pathogenesis of psoriasis. However, human leukocyte antigen (HLA​) susceptibility loci that are significantly related to PsV (HLA‐A1, HLA‐B37, and HLA‐DRw10) were not closely related to pustular psoriasis, indicating that these may be two different diseases. In recent years, IL‐36RN gene mutations have been found in several familial and sporadic cases in Africa, Europe, and Asia.^13^, ^14^, ^15^ This new discovery indicates that a homozygous or compound heterozygous mutation in the gene encoding IL‐36RN may cause GPP, at least in some patients.

This is an important study that investigated the clinical course and prognosis of GPP. We report 110 cases of von Zumbusch type GPP in the past 6 years at our hospital. This disease has a good prognosis if patients are treated correctly and in time. Patients with a history of plaque psoriasis had a greater risk of recurrence than patients with no history of plaque psoriasis. Acitretin and biological agents are good treatment options. For patients with poor economic conditions, acitretin can be used as maintenance therapy. For patients with better economic conditions, biological agents can be used as maintenance therapy. These results will help us to better understand the clinical course of GPP, which is not fully appreciated, and aid in treating GPP. However, this study has some limitations. For example, it was neither a multicentric nor a prospective study and was missing follow‐up data.

## CONFLICT OF INTEREST

None.

## References

[1] Popadic S, Nikolic M. Pustular psoriasis in childhood and adolescence: a 20‐year single‐center experience. Pediatr Dermatol. 2014;31:575–9. 24602000 10.1111/pde.12296

[2] Wang Q, Liu W, Zhang L. Clinical features of von Zumbusch type of generalized pustular psoriasis in children: a retrospective study of 26 patients in southwestern China. An Bras Dermatol. 2017;92:319–22. 29186241 10.1590/abd1806-4841.20175536 PMC5514569

[3] Tay YK, Tham SN. The profile and outcome of pustular psoriasis in Singapore: a report of 28 cases. Int J Dermatol. 1997;36:266–71. 9169323 10.1046/j.1365-4362.1997.00170.x

[4] Baker H, Ryan TJ. Generalized pustular psoriasis. A clinical and epidemiological study of 104 cases. Br J Dermatol. 1968;80:771–93. 4236712 10.1111/j.1365-2133.1968.tb11947.x

[5] Jin H, Cho H‐H, Kim W‐J, Mun J‐H, Song M, Kim H‐S, et al. Clinical features and course of generalized pustular psoriasis in Korea. J Dermatol. 2015;42:674–8. 25819215 10.1111/1346-8138.12863

[6] Youn JI, Oh JG. The clinical study of generalized pustular psoriasis. Kor J Dermatol. 1996;34:233–9.

[7] Xiao T, Li B, He CD, Chen HD. Juvenile generalized pustular psoriasis. J Dermatol. 2007;34:573–6. 17683391 10.1111/j.1346-8138.2007.00334.x

[8] Jiayong TENG. Clinical analysis of 100 cases of psoriasis treated by acitretin. J Leprosy China. 2007;23:858.

[9] Morita A, Yamazaki F, Matsuyama T, Takahashi K, Arai S, Asahina A, et al. Adalimumab treatment in Japanese patients with generalized pustular psoriasis: results of an open‐label phase 3 study. J Dermatol. 2018;45:1371–80. 30302793 10.1111/1346-8138.14664 PMC6585693

[10] Quaranta M, Knapp B, Garzorz N, Mattii M, Pullabhatla V, Pennino D, et al. Intraindividual genome expression analysis reveals a specific molecular signature of psoriasis and eczema. Sci Transl Med. 2014;6:244. 10.1126/scitranslmed.3008946 25009230

[11] Guenova E, Skabytska Y, Hoetzenecker W, Weindl G, Sauer K, Tham M et al. IL‐4 abrogates T(H)17 cell‐mediated inflammation by selective silencing of IL‐23 in antigen‐presenting cells. Proc Natl Acad Sci USA. 2015;112:2163–8. 25646481 10.1073/pnas.1416922112 PMC4343151

[12] Song HS, Kim SJ, Park TI, Jang YH, Lee ES. Immunohistochemical comparison of IL‐36 and the IL‐23/Th17 axis of generalized pustular psoriasis and acute generalized exanthematous pustulosis. Ann Dermatol. 2016;28:451–6. 27489427 10.5021/ad.2016.28.4.451 PMC4969474

[13] Farooq M, Nakai H, Fujimoto A, Fujikawa H, Matsuyama A, Kariya N, et al. Mutation analysis of the IL36RN gene in 14 Japanese patients with generalized pustular psoriasis. Hum Mutat. 2013;34:176–83. 22903787 10.1002/humu.22203

[14] Marrakchi S, Guigue P, Renshaw BR, Puel A, Pei X‐Y, Fraitag S, et al. Interleukin‐ 36‐receptor antagonist deficiency and generalized pustular psoriasis. N Engl J Med. 2011;365:620–8. 21848462 10.1056/NEJMoa1013068

[15] Sugiura K, Takemoto A, Yamaguchi M, Takahashi H, Shoda Y, Mitsuma T, et al. The majority of generalized pustular psoriasis without psoriasis vulgaris is caused by deficiency of interleukin‐36 receptor antagonist. J Invest Dermatol. 2013;133:2514–21. 23698098 10.1038/jid.2013.230

